# Drought Stress Tolerance in Vegetables: The Functional Role of Structural Features, Key Gene Pathways, and Exogenous Hormones

**DOI:** 10.3390/ijms241813876

**Published:** 2023-09-09

**Authors:** Kumail Abbas, Jingrui Li, Binbin Gong, Yusong Lu, Xiaolei Wu, Guiyun Lü, Hongbo Gao

**Affiliations:** Key Laboratory of North China Water-Saving Irrigation Engineering, Ministry of Education of China-Hebei Province Joint Innovation Center for Efficient Green Vegetable Industry, College of Horticulture, Hebei Agricultural University, Baoding 071000, China

**Keywords:** drought stress tolerance, vegetables, structural changes, metabolism, responsive genes, signal transduction, exogenous hormones regulation

## Abstract

The deleterious effects of drought stress have led to a significant decline in vegetable production, ultimately affecting food security. After sensing drought stress signals, vegetables prompt multifaceted response measures, eventually leading to changes in internal cell structure and external morphology. Among them, it is important to highlight that the changes, including changes in physiological metabolism, signal transduction, key genes, and hormone regulation, significantly influence drought stress tolerance in vegetables. This article elaborates on vegetable stress tolerance, focusing on structural adaptations, key genes, drought stress signaling transduction pathways, osmotic adjustments, and antioxidants. At the same time, the mechanisms of exogenous hormones such as abscisic acid (ABA), jasmonic acid (JA), salicylic acid (SA), and ethylene (ET) toward improving the adaptive drought tolerance of vegetables were also reviewed. These insights can enhance the understanding of vegetable drought tolerance, supporting vegetable tolerance enhancement by cultivation technology improvements under changing climatic conditions, which provides theoretical support and technical reference for innovative vegetable stress tolerance breeding and food security.

## 1. Introduction

Vegetables are essential in human diets, offering antioxidants, vitamins, and dietary fibers, complemented by unique flavors, textures, and cultural value [1,2]. Global vegetable production has surged by 65% from 2000 to 2019. Most vegetables contain more than 90% water, so drought stress is a significant impediment, constraining growth, development, and yield [3,4]. In many years, vegetables have evolved multifaceted defense mechanisms to cope with drought stress and maintain growth and metabolism [5]. However, vegetable responses to drought stress vary greatly based on species, stress severity, growth stage, and vegetable parts. Moreover, each vegetable has its own critical stages of water requirement; if water is scarce during these periods, it can lead to a significant reduction in yield and quality (Table 1). Likewise, natural acclimatization also helps vegetables endure diverse environmental constraints; these strategies might fall short of mitigating the rapid impact of drought stress. Under drought stress, vegetables can produce reversible and irreversible physiological and biochemical changes [6]. Therefore, the improvement of vegetable drought tolerance is also multifaceted, and innovative cultivation methods and exogenous regulatory technology are required to meet the normal growth and development needs of global vegetables [5,6,7].

Drought lead to a significant decline in vegetable quantity and quality which ultimately affects food security. Most vegetables suffer sensitivity to drought at around the threshold of ~20% water content [7]. A water deficiency triggers osmotic, ionic, and oxidative stress, closing stomata for a short time and eventually causing vegetables to shrink. This limits CO_2_ uptake, impairs carboxylation, increases photorespiration, and enhances oxidative damage to organelles due to the increased reactive oxygen species (ROS) under drought stress [8,9,10]. The present review further examines how morphological structural attributes influence vegetables’ response to drought. In addition, it also describes the critical genes, signaling transduction pathways, osmotic adjustments, antioxidants, and the role of hormones in managing drought stress tolerance, supporting water conservation and efficient vegetable production strategies. Despite the extensive research on plant drought tolerance already present in the literature, this review fills current gaps in understanding how vegetables respond to and tolerate drought. These insights provide support to enhance vegetable tolerance by cultivation technology improvements and stress tolerance breeding.

**Table 1:** The critical stages of water requirement for different vegetables.

Vegetable Crop	Critical Stage of Water Requirement	Effect of Drought	Reference
Leafy vegetables	During the process of plant growth and development.	Leaf toughness, inadequate foliage development, and nitrate accumulation.	[9]
Potato	The process of tuber formation and the growth of tubers.	Inadequate tuber development and low yield, along with tuber splitting.	[11,12]
Pea	The process of flower formation and the filling of pods.	Decreased root nodulation and stunted plant growth, along with inadequate grain filling.	[13]
Lettuce	Consistently throughout the entire developmental process.	Leaf toughness, inadequate growth of plants, and tip burn.	[14]
Melons	The process of flowering and uniform fruit development throughout.	Muskmelons exhibit diminished fruit quality due to reduced total soluble solids (TSS), decreased sugar and ascorbic acid levels, and increased nitrate content in watermelon fruits.	[15]
Okra	Flowering and pod development.	Intensive decrease in the yield, fiber development, and potential infestation by mites.	[16]
Onion	Bulb enlargement and bulb formation.	Splitting and doubling of the bulb decrease the shelf life.	[17]
Cucumber	Across the flowering period and development of fruits.	Deformed and less vigorous pollens, bitterness in taste, and abnormal fruit shape and size.	[9]
Turnip, carrot, and radish	Development of roots.	Poor and distorted growth of roots, the production of harmful nitrates, and ultimately pungent odor of carrots.	[9]
Cabbage and cauliflower	Formation and enlargement of the head.	Tip burning of stiff leaves; browning and buttoning in cauliflower curd.	[15]
Eggplant	Flower development and fruit setting.	Poor development of fruit color with reduced yield.	
Chili and Capsicum	Development of fruits and fruit setting.	Shedding of juvenile flowers and fruits and reduced dry matter production and nutrient uptake.	[15]
Tomato	Period of flowering and fruits’ rapid enlargement.	Flower shedding hindered fertilization and decreased the size of fruits, and splitting disorders were attributed to calcium deficiency.	[18]

## 2. Drought Stress Impact on Morphological Traits in Relation to Tolerance in Vegetables

Drought stress prompts swift external and internal changes in vegetables, often leading to growth slowdown and potential loss. Research shows that vegetables use phenotypic plasticity to adapt, developing specific traits for drought tolerance. This adaptation is clear in drought-tolerant vegetables with specialized leaves, stems, roots, and cuticles suited for arid conditions. Such evolutionary adjustments enhance growth and early-stage drought tolerance in vegetables by efficiently extracting water from shallow soil layers, minimizing evaporation and boosting water retention intensively.

### 2.1. Drought Stress Impact on External Features in Relation to Tolerance

Unlocking life, water is crucial for seed germination. Drought stress can impede seed imbibition and hinder germination, even when other conditions are favorable [19]. Additionally, it diminishes seedling vitality by compromising water uptake, further impacting germination. During the initial phases of vegetable development, water deficit stress becomes apparent through diminished seed germination and sprouting, resulting in inadequate seedling establishment [20]. In the meanwhile, seeds can exhibit dormancy to delay germination until conditions improve. Drought-responsive genes play a crucial role in sensing stress and regulating hormonal pathways like that of ABA, which inhibits germination. As water availability decreases, ABA levels rise, restraining enzymes responsible for breaking dormancy and promoting germination. Once stress lessens, ABA levels drop, permitting germination-promoting hormones like gibberellins to activate and initiate growth. This mechanism ensures that germination occurs when conditions are favorable for seedling survival, optimizing resource utilization. It has been revealed that peas (*Pisum sativum* L.) subjected to drought stress displayed reduced seedling germination [21]. The combination of low soil water content and other environmental factors can disrupt germination success. Each seed has specific optimal soil moisture levels and temperatures conducive to germination [22,23].

Drought stress during the vegetative phase manifests *itself with* distinct symptoms, including reduced plant height, leaf area, and leaf number and area alterations. Vegetables plant height, a parameter profoundly influenced by drought, is closely associated with cell enlargement and leaf senescence [24]. The decrease in height results mainly from reduced cell expansion, increased leaf shedding, and impaired mitosis due to drought. Previously, numerous studies on vegetable crops, including cabbage (*Brassica oleracea* var. capitata) [25], amaranth (*Amaranth tricolor*; *Amaranth cruentus*) [26], cassava (*Manihot esculenta*) [27], andtomato (*Solanum lycopersicum* L.) [28], indicated a significant reduction in plant height upon exposure to drought. Alongside plant height changes, various organs exhibit notable morphological variations. Leaves, pivotal for assimilation and transpiration, respond to water deficit by adopting smaller leaf areas, increasing leaf thickness, and through enhanced tissue density [29]. Among these shifts, alterations in leaf area crucially impact photosynthesis and yield, making it easily observable in leaves. This transformation in the leaf area is a result of factors such as leaf turgor pressure, canopy temperature, and photo-assimilate availability [30].

With regard to morphological responses, previous studies on vegetable crops have consistently found a notable reduction in leaf dimensions to tolerate water deficit, including width and length [31,32,33,34,35]. Plant leaves are closely linked to biomass, suggesting that leaf numbers influence total biomass under drought conditions. In a study presented by Paim et al., watering at 90% and 80% field capacity, followed by a 4-day irrigation pre-harvest pause, increased carotenoid content and biomass [14]. The findings demonstrated that fresh weight of a given crop was significantly affected under drought stress conditions compared to a control group [36]. In such scenarios, vegetable crops require a robust root system to anchor themselves and extract water and nutrients from their surroundings. This ensures their survival and growth even under water-scarce conditions, heightening the critical role of root structures in enhancing drought tolerance.

Apart from leaves, vegetable roots, which are directly responsible for water uptake, play a crucial role in drought stress [37]. Developed root systems improve water uptake from soil reserves, aiding vegetables’ survival in arid conditions. Published research underscores water as the primary environmental factor influencing root development, particularly relevant in arid contexts where root morphological adaptations are critical [38]. Root attributes like density, branching, and root hair distribution have a profound impact on vegetables’ ability to cope with water stress. Drought stress inhibits seedling development, leading to elongation and thinning of fine roots, shortened lifespan of fine roots with different diameters, heightened elongation of root hairs, and accelerated root decay [39]. Prior studies reveal that selected vegetable crops adapt to drought by boosting root complexity and elongation and reducing branching angles, leading to deeper and more resilient root systems. Similarly, drought-treated plants optimize water uptake by minimizing lateral root branching, prioritizing axial root elongation and deeper rooting. Water availability also influences root distribution; soybean (*Glycine max*), field pea (*Pisum sativum* subsp. Arvense), and chickpea (*Cicer arietinum*) display sensitivity, exhibiting a higher root/shoot ratio due to decreased biomass relative to roots [40]. Drought stress additionally impacts plants’ external morphology, with a 39.02% average internode length increase post-drought treatment during early vegetative stages, potentially compromising complete root structure [41].

### 2.2. Drought Stress Impact on Internal Features in Relation to Tolerance

In addition to altering external morphology, vegetables suffering from water deficit also experience changes in internal structure. The leaf epidermis forms a cuticle, a lipid membrane that acts as a barrier, limiting water loss and enhancing drought tolerance. A thicker cuticle boosts energy reflection, reduces transpiration, and improves drought resilience. Treatment for dehydration significantly increases cuticular lipid content, particularly wax alkane content, and augments the amount of cutin monomers, leading to thicker cuticles with increased osmiophilicity [42]. Similarly, leaves enhance drought tolerance by increasing wax coverage, cuticle thickness, and osmiophilicity [43]. Wu et al. [44] and Liu et al. [45] observed that introducing orange *CsECR* in transgenic tomato (*Solanum lycopersicon*) plants elevated total and aliphatic wax content, reducing cuticle permeability in leaves and fruits. Structural adjustments include elevation of mesophyll palisade tissue levels, reducing spongy tissue, increasing cell layers, and decreasing intercellular space to adapt to drought stress [46]. Stomatal development is vital; drought stress heightens stomatal length, width, density, and opening, with reduced density enhancing stress tolerance [47]. Over time, leaf epidermal cells undergo expansive changes, and both epidermal and spongy tissue cell walls thicken during drought stress. Prolonged stress leads to compressed, spongy tissue cells filled with sclerenchyma [48]. Lignification and channeling tissue degree of the epidermis significantly affect plant drought resistance. Water-deficient plants exhibit lower leaf lignin levels than well-watered counterparts [49]. The xylem of stems and roots thickens in stress-treated plants. Drought stress reduces vessel diameter but increases root vessel number and diameter [50]. Root system architecture, encompassing factors like root angle, primary and lateral root number/length, and root hair density/length [51,52,53], is influenced by phytohormones such as ABA, auxins, cytokinin, ethylene, and jasmonic acid. Drought-induced changes often lead to increased lateral root and root hair generation [54]. In a recent study, topical application of melatonin to tomato plants under water-stressed conditions was found to significantly improve root architecture [53]. Together, these adaptations help vegetables to withstand water scarcity.

## 3. Drought Stress Impact on Physiological and ROS Metabolism in Relation to Tolerance in Vegetables

### 3.1. Drought Stress Impact on Physiological Response in Relation to Tolerance

When vegetables are exposed to drought stress, they exhibit diverse defense mechanisms, influenced by species and drought stress intensity and duration [27]. Many studies have investigated how drought stress impacts physiological parameters and the quality of vegetables (Table 2). To measure drought stress, several key physiological parameters serve as indices of water availability. These include leaf water potential, the maximum quantum yield of PSII (Fv/Fm), water-use efficiency (WUE), cell membrane integrity, relative water content (RWC), and osmotic adjustment (OA) [55,56]. Drought stress reduces leaf water potential, driven by diminished cell turgor pressure and related processes, including stomatal closure. This response curtails water losses and limits nutrient uptake from the soil [57,58]. In tomato (*Solanum lycopersicum*), stomata close at a water potential of around −0.7 to −0.9 MPa, while in pepper (*Capsicum annum* L.), this closure range is slightly higher, at −0.58 to −0.88 MPa [59]. While stomatal closure mitigates transpiration, it also reduces gas exchange and the rate of photosynthesis. Prolonged drought stress has further impacted on biochemical processes such as carboxylation efficiency, Rubisco regeneration, Rubisco quantity, and PSII activity inhibition. In contrast, drought-tolerant species maintain carbon fixation during stress, owing to high WUE and the ability to promptly reopen stomata when the water deficit subsides. WUE is the ratio of dry matter accumulation to water consumption during the growing season, or it can also be expressed as the ratio of photosynthesis to transpiration over a specific time frame [60]. WUE reflects a genotype’s capacity to effectively extract water from soil in water-stressed environments [59]. Variations in WUE among genotypes stem from their ability to channel soil water towards assimilation instead of transpiration, potentially differentiating drought-tolerant from susceptible genotypes. Breeding plants for high water-use efficiency has often resulted in slow growth and is less appealing agriculturally. For example, enhancing water-use efficiency increased yields by 15% under drought, but this benefit diminished with 400 mm rainfall, nullifying the yield increase [61]. This intricate interplay underscores how plants strategically respond to drought, emphasizing the importance of these physiological parameters in assessing and understanding their adaptation mechanisms [58,59]. Expanded changes in WUE have been demonstrated in cowpea cultivars, soybean, Amaranthus sp., and tomato plants [62,63,64]

Amid drought stress, photosynthesis slows due to incomplete conversion of captured light into chemical energy, with excess energy causing photoinhibition reduction in maximum quantum yield of PSII (Fv/Fm). In a study on wild asparagus (*Asparagus acutifolius*), Mantovani et al. [65] observed that soil water content and leaf water potential decreased as water stress increased over six days. The decrease in leaf water potential led to a significant reduction in net photosynthesis. Various mechanisms mitigate photoinhibition’s impact, including non-photochemical quenching, photorespiration via the Mehler reaction, energy dissipation, and chlorophyll regulation. Fv/Fm serves as both a water stress indicator and a discriminator between water stress-tolerant and sensitive genotypes. For instance, drought-tolerant tomato genotypes exhibit preserved PSII activity and higher photosynthetic efficiency under water stress than susceptible genotypes [59]. It has been demonstrated in lettuce (*Lactuca sativa* L.) that drought’s impact on chlorophyll florescence parameters vary depending on the treatment time. In general, under water deficit, there was a decrease in PSII efficiency and parameters related to photochemical quenching, while non-photochemical parameters showed a tendency to increase [66,67,68]. Lettuce plants (*Lactuca sativa* L.) subjected to varying levels of water saturation were examined, with fully irrigated plants (100% irrigation) being compared to those experiencing moderate water stress (90% and 80% of total irrigation, with irrigation halted four days before harvesting). Surprisingly, the plants undergoing moderate stress displayed improved quality characteristics compared to the fully irrigated control. Specifically, at 80% water supply, these plants exhibited the highest levels of carotenoids (2.74 µg g^−1^) and chlorophyll contents both at the start (15.69 µg g^−1^) and after 7-day storage at 4 °C (18.24 µg g^−1^) [14]. This highlights Fv/Fm’s utility in assessing water stress and genotype responses, establishing a crucial link to plant adaptation strategies in drought.

**Table 2:** Vegetable species under drought stress: physiological and ROS metabolite aspects.

Vegetable Crops and Cultivation Condition	Drought Stress Treatment	Impact on Crop and Drought Stress Tolerance	References
Potato (*Solanum tuberosum* L. cultivars) in greenhouse	Irrigation interruption for 12–13 days before tuber formation.	Decrease in: relative water content (RWC); leaf osmotic potential. Elevation of: nitrogen (N) levels and augmented levels of proteins; proline within the leaves.	[69]
Lettuce (*Lactuca sativa* L.) Veneranda cultivar in greenhouse	Watering at 90% and 80% field capacity, followed by a 4-day irrigation pause before harvest (inducing acute stress).	Increase in: carotenoids; biomass; chlorophyll content; flavonoids; phenolic acids.	[14]
Lettuce (*Lactuca sativa* L.) butterhead (Aquino) and red butterhead (Barlach) cultivar in greenhouse	Soil water contents of 70% and 40%	Reduction in PSII efficiency; elevated biomass.	[70]
Eggplant (*Solanum melongena* L.) field	Seven regimes of irrigation.	Reduction in: fruit weight and firmness; total sugars; proteins. Increase in: CAT and APX activity; total phenols; flavonoids.	[71]
Amaranth (*Amaranth tricolor*; *Amaranth cruentus*) in greenhouse	Suspension of watering for 14 days.	Reduction in: plant height, leaves, roots, stem fresh and dry weight; leaf area; chlorophyll content. Increase in transpiration efficiency.	[72]
Wild asparagus (*Asparagus acutifolius* L.) in greenhouse	Leaf water potential of −1.4 MPa and −2.4 MPa over 6 days.	Decrease in net photosynthesis.	[65]
Common chicory (*Cichorium intybus* L.) in greenhouse	80%, 60%, and 40% of field capacity.	Increase in: SOD and CAT activity; proline and ascorbic acid content; abscisic content in leaves.	[73]
Cassava (*Manihot esculenta* Crantz), cv. SC205, GR4, RS0I, and SC124 in glasshouse	50% and 20% of field capacity.	Reduction in: chlorophyll content and RWC and plant height. Increase in: H_2_O_2_; malondialdehyde (MDA), ascorbic acid; glutathione; SOD and CAT activity; total phenols. Overexpression of Mn-SOD, CAT, and GR genes.	[27]
Cabbage (*Brassica oleracea* var. *capital*) in greenhouse	80% and 60% of the field capacity.	Increase in: H_2_O_2_, lipid peroxidation, electrolyte leakage, proline content, and sucrose. Reduction in: biometric parameters (plant height, stem diameter, number of leaves, leaf area, fresh and dry shoot weights); photosynthesis; stomatal conductance and transpiration and chlorophyll content.	[25]
Tomato (*Solanum lycopersicum* L., cv. landrace Cietttaicale and Moneymaker) in growth chamber	Treatment irrigation with 50% of the field capacity every 48 days for twenty days.	Reduction in: osmotic potential, stomatal conductance, photochemical efficiency of PSII, leaf starch. Increase in: non-photochemical fluorescence quenching; ABA and IAA contents in leaves and roots; soluble sugars; lipid peroxidation; proline and antioxidant activity in roots.	[28]
Pepper (*Capsicum annum* cultivars (Nongchengjiao-2 and Shansshu-2001)) in greenhouse	Grown under four water regimes: 80, 60, 40, and 20 of field capacity for 6, 12, 18, and 24 days.	Reduction in RWC; increased proline content, total soluble proteins, and SOD, POD, and CAT activity at the onset of stress; decreased leaf area and fruit yield.	[74]
Sage (*Salvia officinalis*) in field	Stop irrigation for six weeks.	Hampers stomatal closure; reduction in CO_2_ assimilation; increase in NADPH.	[75,76,77]
Pepper (*Capsicum chinense*) (cultivars. Rex and Genesis), *Capsicum annum* cv. Padron)) in greenhouse	Restriction of water during the flowering stage for 7, 10, 14, 18, and 21 days.	Noticeable decrease in RWC, along with an increase in electrolyte leakage and proline content.	[57]
Soya bean (*Glycine max* L.) in field	Treatments applied to control drought at different reproductive phases.	Drought reduces the seed germination.	[78]
Okra (*Abelmoschus esculentus* L. Moench) in field experiment	Exposed to water deficit under various waters regimes for 5 or 10 days.	Waters restrictions exceeding ten days during the reproductive period result in diverse growth and yield effects.	[79]

Under drought stress, cell membrane integrity can be disrupted, causing changes in permeability and consequent ion loss. Electrolyte leakage (EL), measured by the release of ions from cells, indicates this disruption. Notably, cytoplasmic electrical conductivity is a marker for drought tolerance, where tolerant genotypes exhibit minimal electrolyte leakage due to preserved membrane integrity, contrasting with susceptible genotypes [4]. Furthermore, RWC measures plant tissue water status during water stress. The specific genotype influences its decrease with increasing water deficit [27]. Numerous studies have examined how drought stress impacts physiological parameters and vegetable quality. Escalante-Magana et al. [57] investigated three pepper (*Capsicum chinense*) varieties under greenhouse conditions, subjecting them to 7 to 21 days of water deficit. As stress intensified, all varieties decreased their RWC from 85.0% to 32.6% by day 21. Notably, all stress-treated plants displayed robust recovery capacities, maintaining RWC-like controls. Despite recording a high EL of 93% by day 21 and a steady increase in proline content, these pepper cultivars exhibited resilience to water stress [74], aligning with findings in other crops like cabbage (*Brassica oleracea* var. *capitata*) [25], tomatoes (*Solanum lycopersicum* L.) [28], and various potato (*Solanum tuberosum* L.) cultivars [69]. To achieve efficient irrigation, one should select drought-resistant varieties, improve soil quality, regulate watering times, prune for reduced water demand, and closely monitor plant health.

Plants counter drought-induced physiological damage by adjusting osmotic adjustment (OA), accumulating organic solutes to lower cellular osmotic potential, and allowing water influx to restore turgor. High OA values in Brassica species aid water extraction from deeper soil layers (90–180 cm) and maintain turgor even at low leaf potentials (−2.4 MPa) [59]. Common osmolytes under water deficit include proline, glycine betaine, sugars, polyols (sorbitol, mannitol, etc.), and low-molecular-weight compounds like dimethyl sulfoniopropionate (DMSP) [80]. These osmolytes enable water absorption and stabilize proteins, cell membranes, chloroplasts, and liposomes against stress damage [7]. A high osmotic potential value of Brassica species facilitates water extraction from deeper soil layers, typically within the 90 to 180 cm range [59]. Proline, notable for scavenging radicals, providing nitrogen and energy, and aiding cell wall protein synthesis, plays a multifunctional role in stress responses [81]. Glycine betaine accumulates in diverse organisms under stress and is a protective molecule [57]. Drought stress impacts numerous physiological and biochemical processes vital for plant growth and development. Inadequate water supply during critical growth stages like flowering and fruit setting profoundly jeopardizes vegetable yield and quality [9].

### 3.2. Drought Stress Impact on ROS Metabolism in Relation to Tolerance

**Figure 1:**
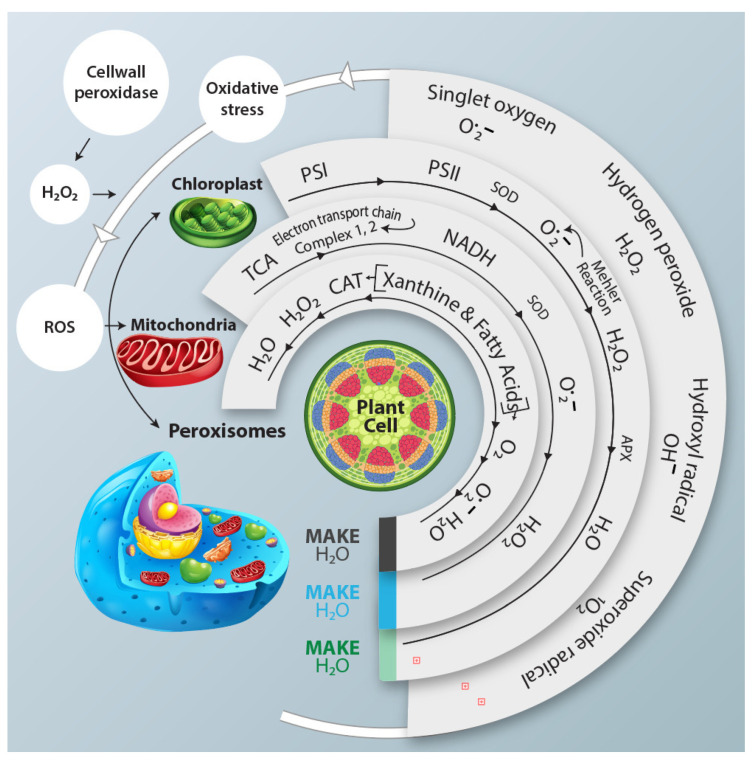
This schematic representation illustrates the response of different cellular organelles, including chloroplast and peroxisomes, to the generation of the ROS and their subsequent neutralization by antioxidant enzymes under water deficit conditions. ROS Pathways are activated in each organelle, while antioxidant enzymes counteract their potential harmful effect. NADH: Nicotinamide adenine dinucleotide (cofactor).

Water scarcity causes stomatal closure in vegetables, escalating the production of ROS within organelles, including singlet oxygen (O_2_^•−^), hydrogen peroxide (H_2_O_2_), hydroxyl radical (OH^•^), and superoxide radical (^1^O_2_). Under drought stress, ROS act as the signaling molecules and trigger programmed cell death pathways through oxidative stress-induced cascades and gene activation, leading to controlled degradation of cell compartments (Figure 1). Increased ROS generation leads to oxidative stress, detrimentally impacting plant growth and productivity [82]. Major metabolic hubs such as chloroplasts, mitochondria, and peroxisomes promote ROS accumulation via the Mehler reaction, electron transfer, and photorespiration. ROS trigger oxidative damage to chloroplasts, diminishing carboxylation [83,84]. Smaller leaf size additionally curtails carboxylation. Poor regulation of acyclic electron transport hampers ATP synthesis. These factors collectively contribute to a notable decline in vegetable photosynthesis. Under water deficit conditions, capacity to endure water scarcity stress and sustain water potential is compromised. Given their succulent nature, vegetables generally contain over 90% water, rendering them susceptible. Precise regulation and effective metabolism of intracellular ROS production and elimination are crucial in preventing cellular component damage and uphold growth, metabolism, development, and overall plant productivity. To counteract ROS-induced harm, plants synthesize various antioxidative enzymes alongside osmolytes such as proline and glycine betaine [82,85]. SODs principally tackle ROS, converting ^1^O_2_ into H_2_O_2_, while CATs convert H_2_O_2_ to H_2_O, aided by APXs using ascorbate as a specific electron donor [7]. Vegetables elevate antioxidant enzymes and osmolyte production under drought stress. Drought-tolerant genotypes generally exhibit higher SOD, POD, CAT, APx, GR, proline, and glycine betaine levels than sensitive genotypes, although variations exist among vegetable types. Tomato plants, for instance, exhibit heightened antioxidant activity when exposed to drought [86]. The outcomes in chicory (*Cichorium intybus* L.) plants indicated distinct responses among ecotypes. Remarkably, the Siyah Shiraz ecotype exhibited a notably enhanced defense mechanism compared to the other ecotypes. This superiority was evident regarding higher levels of antioxidant enzymes [73]. To mitigate drought stress and bolster antioxidative enzyme activity, approaches like nano-organic fertilizers, foliar mineral application [87], and grafting techniques have been explored [88]. Drought-tolerant eggplant and sweet pepper genotypes displaying robust antioxidant activity demonstrate efficient drought tolerance during seedling stages [89,90,91,92,93,94]. Similar responses were observed in cucumber seedlings subjected to water deficit conditions, where SOD, POD, and CAT production increased [95,96]. In various plant species such as green bean (*Phaseolus vulgaris* L.) [97], pea [98,99], soybean (*Glycine max* L.) [100], and chickpea (*Cicer arietinum* L.) [101], the concentrations of antioxidants have been noted to exhibit a more substantial increase during the recovery phase following oxidative stress, as opposed to the stress phase itself.

Similarly, drought stress resulted in decreased height of eggplant (*Solanum melongena* L.) plants [71]. Water deficit influences external features like weight, size, and firmness of the fruits and internal characteristics such as total sugars and proteins. Notably, water stress led to heightened levels of total phenols, flavonoids, and antioxidants, along with increased APX activity. In drought stress, resistant common bean and horse gram cultivars have exhibited heightened activities of critical antioxidants, including SOD, APX, GR, GST, GPX, and POD [102,103]. In conclusion, increased antioxidant activities within vegetables are crucial for enhancing drought tolerance by protecting against oxidative stress.

## 4. Signaling Transmission and Transduction in Vegetable Plants under Drought Stress

Vegetables plants use a three-step signaling mechanism to respond to environmental stimuli. First of all, sensory cells perceive stimuli, react to them, and produce intercellular messenger substances. These messengers then travel between cells or tissues and affect the locations of the receptor cells. These receptors are proteins found in the plasma membrane that bind to and interact with external substances known as ligands or elicitors. Finally, the acceptor cells undergo transduction and respond accordingly [104].

### 4.1. Signal Transmission in Vegetable Plants under Drought Stress

**Figure 2:**
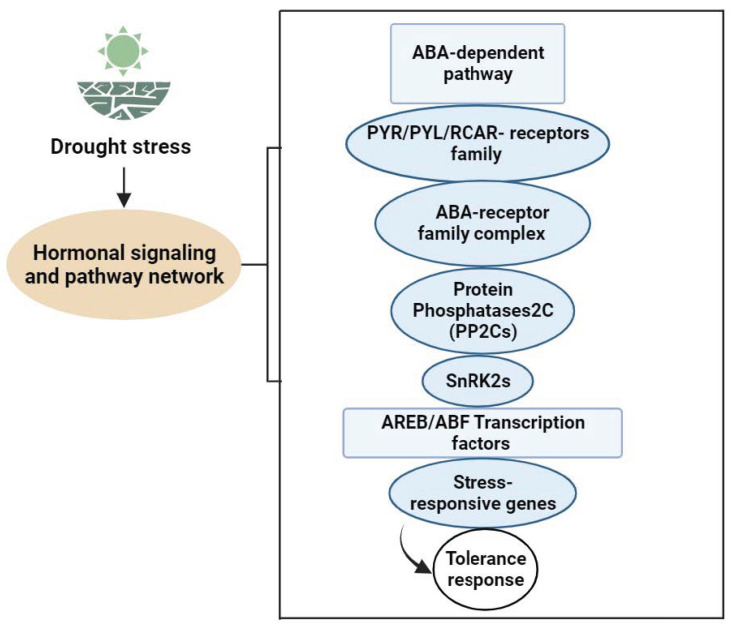
ABA signal transduction pathway network in Vegetable Plants under Drought Stress.

Limitations of soil moisture affect leaf hydration and physiology, with leaf water potentially signaling stress. Leaf water potential and turgor pressure influence ABA and cytokinin production, transport, and distribution [105]. In drought stress, changes in turgor pressure caused by cell water loss can have an effect on the hydraulic signal. Both hydraulic and electrical signals are crucial for drought-related signal transduction. ABA-mediated responses in protective vegetable cells support its pivotal role in drought signaling, encompassing hydraulic and non-hydraulic signals [106,107]. Fromm et al.identified electrical signals as the communication method between roots and shoots under water deficiency. The osmotic stress signal is transformed into a second messenger via membrane receptors, intensifying signal propagation through downstream effectors. Hormones, Ca^2+^, IP3, phosphatidic acid, and ROS are essential second messengers in the early signaling of drought [108]. Phytohormones serve as chemical signals that regulate growth, transmitting messages across various vegetable parts. They act in low concentrations and facilitate long-distance transmission of signals. These stress-responsive genes’ products not only contribute to plant adaptation and survive severe environmental conditions, but they could also be involved in the production of several phytohormones such as ABA, SA, and ET. These hormones then serve as regulatory molecules, enhancing the first signal and starting a second round of signaling that can follow the same basic pathway or involve multiple signaling components. Drought stress raises these phytohormones’ levels but decreases active substances like cytokinin, indicating negative signals. The lipophilic plant hormone ABA is essential to water stress responses, as it controls growth, germination, aging, drought, salt, and cold adaptation. It is a chemical signal that integrates with roots and travels toward shoots and leaves [109,110]. This hormone orchestrates complex signaling cascades, inducing stomatal closure and activation of the expression of drought-responsive genes, ultimately influencing plant growth and development [106,107]. Importantly, ABA activates kinases, phosphatases, G proteins, and the ubiquitin pathway. Multiple ABA receptors exist: ABAR/CHLH, •GCR2, •GTG1/2, and PYR/PYL/RCAR. These receptors, acting as protein kinases, alter their structure upon ABA binding, influencing downstream signaling proteins for intercellular signal transmission (Figure 2). Research on these receptors continues, as their specific functions remain uncertain. ABAR/CHLH, located in chloroplasts, is a magnesium ion chelatase H subunit involved in chlorophyll synthesis and inter-organelle signal exchange under stress [111,112]. GCR2, a G protein-coupled receptor at the cell membrane, interacts with GPA1, releasing a G protein. The G protein splits into Gα and Gβγ dimers, influencing ABA responses. GTG1/2, identified through bioinformatics, may modulate ABA signaling via GPA1–GTP and GTG–GTP interactions, affecting ABA binding. PYR/PYL/RCAR binds extracellular ABA, inhibiting downstream phosphatase PP2C activity. These receptors play a vital role in ABA-mediated responses; however, there is yet more to be explore about ABA’s detailed mechanisms [113].

### 4.2. Signal Regulation Pathways in Vegetable Plants under Drought Stress

Drought stress signaling in plants involves two pathways: Ca^2+^ -dependent signaling via activating calmodulin-dependent protein kinase (CDPK); and ROS activated by the mitogen-activated protein kinase (MAPK) cascade pathway. Calcium (Ca^2+^) is essential for drought stress signaling, maintaining cell membrane stability, cell wall structure, and intracellular balance in plants. Previous studies reported that extracellular Ca^2+^ promotes intracellular Ca^2+^ content in guard cells via the calcium-sensing receptor (CAS), the first messenger [114]. ABA production closes stomata in drought conditions, increasing cytoplasmic Ca^2+^ as the second messenger in osmotic stress response [115]. Guard cells temporarily elevate Ca^2+^ during drought, closing stomata and improving water-use efficiency. The ABA-dependent Ca^2+^ pathway activates plasma membrane calcium channels and releases intracellular Ca^2+^. High Ca^2+^ levels restrict inward potassium channels, influencing anion channels and causing potassium outflow. Guard cell Ca^2+^ regulation by IP3 and cADPR helps close stomata. Ion interactions from second messengers activate calcium channels, closing stomata. Downstream Ca^2+^ signals protein receptors to stress. Ca^2+^-sensitive promoter elements, calcium-regulated kinase-mediated phosphorylation, and transcription factor-mediated gene expression modulation are fundamental mechanisms [116,117]. Plants use CDPKs, CaM, and CBLs to respond to drought stress and understand specific Ca^2+^ signals.

ROS activated by MAPK cascade pathway-controlled ROS generation activate defense systems against abiotic stress [118]. ROS generated due to drought stress act as signaling molecules in vegetable plants, triggering programmed cell death (PCD) pathways through oxidative stress-induced signaling cascades and gene activation, leading to controlled cellular breakdown and adaptation to stress conditions. Stability and diffusivity make H_2_O_2_ a vital messenger molecule in animal and plant cells among ROS. Peroxisomes and chloroplasts generate H_2_O_2_ quicker than mitochondria during drought stress, although mitochondria are most sensitive to oxidative damage [119,120]. High mitochondrial H_2_O_2_ levels may suggest antioxidant defense intensification or planned cell death under extreme oxidative stress. H_2_O_2_ controls gene expression, protein phosphorylation, and calcium mobilization. Pei et al. [121] linked ABA-induced stomatal closure to H_2_O_2_ and Ca^2+^ channel activation in guard cells. Mori et al. related ROS signaling to stomatal closure [122]. Yan et al. found that ABA induces ROS, signaling stomatal control [123]. H_2_O_2_ also phosphorylates MAPK, which affects downstream gene expression cascades [124].

## 5. Drought Stress Tolerance-Related Functional and Regulatory Genes in Vegetables

**Figure 3:**
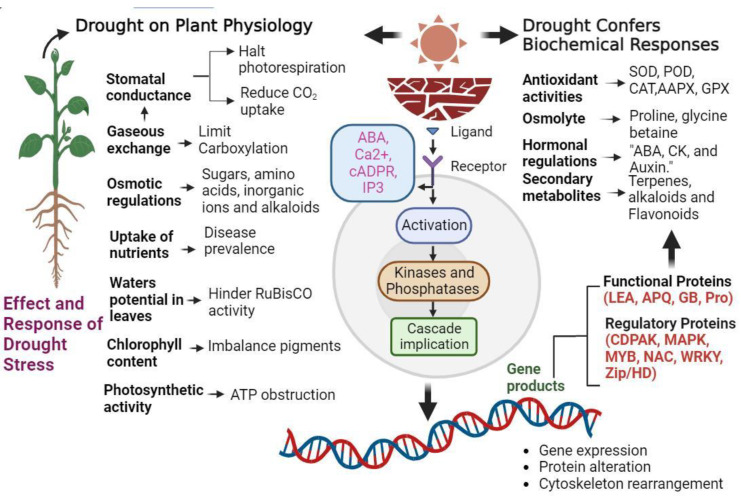
This illustration highlights the complex series of events involved in plant response to drought stress, from signal perception on the cell membrane to the activation of stress-responsive genes and adaptive changes.

Broadly speaking, genes involved in drought stress response can be classified into functional genes that directly counteract environmental stress and regulatory genes that control these responses (Figure 3).

### 5.1. Drought Stress Functional Genes

Enzymes responsible for proline accumulation can be categorized into three groups based on distinct pathways. The first group involves proline synthesis, including ∆-pyrroline-5-carboxylate synthetase (P5CS), pyrroline-5-carboxylate reductase (P5CR), and ornithine-δ-aminotransferase (δ-OAT). The second group pertains to proline degradation, encompassing proline dehydrogenase (ProDH) and △-pyrroline-5-carboxylate dehydrogenase (P5CDH). The third group concerns the proline transport-related enzyme ProT. Proline synthesis in plants occurs in the cytoplasm and chloroplast, involving the glutamic acid (Glu) and ornithine (Orn) synthesis pathways [125]. The Glu pathway is more prevalent under osmotic stress and nitrogen deficiency, whereas the Orn pathway thrives in nitrogen-rich environments [126]. In the Glu pathway, Glu is transformed by ∆-pyrroline-5-carboxylate synthetase (P5CS) into glutamic semialdehyde (GSA). Subsequently, GSA undergoes an automatic cycle, leading to the formation of pyrroline-5-carboxylic acid (P5C), which is converted into proline (Pro) by pyrroline-5-carboxylate reductase (P5CR) [127,128]. In contrast, the Orn pathway employs ornithine (Orn) as the substrate and ornithine-δ-aminotransferase (δ-OAT) as the enzyme. The key substrates and products in both pathways include Glu, Orn, GSA, P5C, and Pro, with essential enzymes such as P5CS, P5CR, and δ-OAT driving the reactions.

La Rosa et al. observed a similar outcome in their study, where overexpression of the soybean *P5CR* gene in tobacco led to a fivefold increase in P5CR activity. However, the proline levels in the transgenic tobacco did not show a significant increase [129]. In contrast, overexpression of the P5CS gene from moth bean (*Vigna aconitifolia* L.) in rice resulted in elevated P5CS enzyme production and proline accumulation [130]. Likewise, transgenic tobacco expressing *AtP5CS* exhibited higher proline content and improved osmotic regulation [131]. Consistent effects were also noted in potato [132], soybean [133], and other crops. Another pivotal enzyme, δ-OAT, exhibited heightened activity under drought conditions [134]. Overexpression of δ-OAT in various crops significantly boosted proline content. Investigations into proline dehydrogenase (PDH1) mutants demonstrated that blocking Pro catabolism allowed plants to sustain growth through active Pro breakdown under low water potential [135]. Moreover, the transport of proline necessitates the involvement of ProT, a member of the amino acid/auxin permease (*AAAP*) gene family in plants. ProT is a typical Na^+^-dependent sub-amino acid transporter that undergoes active transport via proline coupling with the Na^+^ electrochemical gradient. This process requires the participation of Na^+^–K–ATPase [136].

The enzymatic production of glycine betaine (GB) in plants is well understood. Choline is the starting material for GB production, which is enhanced by three adenosine–methionine-dependent phospho-ethanolamine (PE) methylation stages by PEAMT in the cytoplasm [137]. PEAMT has N- and C-terminal methyltransferase domains. The N-terminal domain methylates PE to make phosphate-monomethyl-ethanolamine (P-MME), and the C-terminal domain methylates P-MME to form P-DME, which produces phosphocholine (PC) [138]. It has been shown in previous studies that spinach and tobacco convert PC to choline via different mechanisms through dephosphorylation [139]. A two-step oxidation produces betaine. The rate-limiting phase of GB production involves ferredoxin-dependent choline monooxygenase (CMO) oxidizing choline to betaine aldehyde [140]. The next step includes NAD^+^-dependent betaine aldehyde dehydrogenase (BADH) oxidizing betaine to produce betaine [141]. CMO is a ferredoxin-dependent rate-controlling enzyme with a Rieske-type (2Fe-2s] active site found in chloroplasts and other cellular compartments [142]. Due to diminished ferredoxin from photosynthetic electron transport, light induction can boost CMO activity, which is normally modest and unstable. CMO regulates this process to maintain balance and pace. Betaine aldehyde is harmful to plant cells; therefore, CMO must synthesize enough for betaine synthesis without accumulating too much. BADH, a key betaine synthesis enzyme, is a dimer encoded by a single-chain nuclear gene with two alleles. It is an aldehyde dehydrogenase superfamily member that needs NAD^+^ and NADP^+^ to work. In plants, BADH activity increases with NAD^+^ [143]. Dicotyledonous BADH is mostly present in chloroplast stroma, but monocotyledonous BADH may be found in microsomes. Two BADH isozymes exist, with BADHII being more important [144]. Standard BADH activity is minimal, but stressors like low temperature, dryness, and excessive salinity increase it. This stress-induced BADH activity increases betaine production. Some plant species have been transformed using GB biosynthesis pathway genes to increase abiotic stress tolerance through genomics, proteomics, and genetic engineering. Most genetically altered plants that generate GB do not naturally collect it [145].

Shen et al. isolated and characterized the *CMO* gene from spinach, subsequently transferring it to tobacco, resulting in enhanced resistance to salt and drought [146]. Fan et al. introduced the *SoBADH* gene from spinach into sweet potato, leading to heightened BADH activity and increased tolerance to abiotic stress [147]. Further investigations have also shown that introducing the *CMO* gene into rice and tobacco can significantly enhance their ability to withstand salt and drought stress [148]. Additionally, Li et al. engineered tomatoes with the *SoBADH* gene, resulting in elevated betaine levels and improved resilience to stress [149]. Similarly, Ishitani et al. identified and cloned the *BADH* gene from barley, successfully transferring it to tobacco and enhancing the plant’s drought tolerance [150]. Luo et al. down-regulated the dehydrin gene *CaDHN5* using VIGS in *Capsicum annuum* L. plants and overexpressed *CaDHN5* in Arabidopsis. They observed a positive correlation between *CaDHN5* expression and the genes of manganese superoxide dismutase (Mn-SOD) and peroxidase (POD) [151]. Plant soluble sugar metabolism, exemplified by sucrose, is intricate. In sucrose synthesis, FBPase (fructose-1,6-bisphosphatase) and SPS (sucrose phosphate synthase) are pivotal enzymes. FBPase hydrolyzes fructose-1,6-diphosphate (FDP) to fructose-6-phosphate (F6P), generating sucrose in the cytoplasm and starch in the chloroplast. Overexpressing FBPase decreases soluble sugar levels; its activity in potato reduces sucrose synthesis [152]. SPS converts fructose-6-phosphate and UDP-glucose to sucrose-6-phosphate, then SPP hydrolyzes it to form sucrose. SPS is key in controlling sucrose synthesis due to irreversibility. Introducing *ZmSPS1* into tomato [153], potato [154], and Arabidopsis [155] enhances SPS activity and sucrose levels.

### 5.2. Drought Stress Regulatory Genes

In various species, novel genes have been identified that induce changes in physiological and morphological traits under drought stress. For instance, the regulation of root length and numbers involves multiple genes and dominant allele expression, while root thickness relies on recessive allele expression [156]. Genes linked to solute accumulation, like *mtlD* for mannitol and *P5CS* for proline, contribute to maintaining water potential and entail diverse enzyme-mediated molecule synthesis [157]. Overexpressing these genes in vegetables yields specific responses to drought stress. For example, *ABF4* transcription factor genes not only enhance drought tolerance in potatoes but also improve tuber quality and yield [158]. The *SlGRAS4* transcription factor gene heightens stomatal sensitivity to ABA, curbing water loss [159]. The *AVP1* gene influences root growth [160], the *NADP-Me* gene impacts stomatal conductance and water-use efficiency [161], and the *Wilty* gene participates in tomato leaf wilting under drought stress [156]. Furthermore, essential for plant stress resilience, AP2/ERF transcription factors contribute to drought resistance via diverse pathways. They modulate plant hormone synthesis to regulate drought response. For instance, ERF1, a crucial component of jasmonic acid and ethylene signaling, coordinates abscisic acid signals as well, enhancing drought tolerance by orchestrating stress-related gene regulation, as proposed by Cheng et al. [162]. Similarly, Zhang et al. [163] demonstrated that *JERF1* overexpression enhances drought tolerance in transgenic plants. They revealed that *JERF1* triggers the activation of key ABA synthesis enzymes *OsABA2* and *Os03G0810800*, elevating ABA content. This implies that JERF1’s role in drought response might be mediated via the ABA pathway. AP2/ERF transcription factors govern wax synthesis, which helps plants resist drought. Wang et al. found that OsWR1 interacts with DRE and GCC boxes in wax-associated gene promoters *OsLACS2* and *OsFAE1-L*. This direct regulation changes gene expression, affecting wax formation by adjusting long-chain fatty acids and alkanes. Thus, overexpressing *OsWR1* boosts drought resistance [164]. Chen et al. found that *MdMYB46* enhances secondary cell wall biosynthesis and lignin deposition by binding to lignin-related gene promoters. It also boosts osmotic stress tolerance through direct activation of stress response signals [165]. Findings by Geng et al. showed that under drought conditions, MdMYB88 and MdMYB124 regulate root xylem development and control cellulose and lignin accumulation by directly binding to *MdDVND6* and *MdMYB46* promoters [166]. In response to drought stress, GhWRKY17 modulates the ABA signaling pathway and triggers ROS production within plant cells [167]. In conclusion, while enhancing the expression of stress-related genes can lead to improved resistance to various challenges, it is important to note that excessive expression of such genes could have adverse impacts on plant growth and development. For instance, transgenic plants with heightened *OsNAC6* expression exhibited enhanced resistance against drought, but they also displayed negative effects like stunted growth and reduced yield [168].

## 6. Exogenous Hormonal Regulation in Enhancing Vegetable Drought Stress Tolerance

Vegetables’ drought tolerance can be improved by using natural and synthetic plant growth regulators. Plant growth and environmental stress response depend on phytohormones. Specific cells are genetically predisposed to respond to hormones during certain plant life cycle periods. At certain times and places in their life cycle, vegetables need hormones, which must dissolve when no longer needed. These hormones influence vegetables’ responses, including growth patterns and physiological modifications necessary for water-deficient vegetable growth. Phytohormones regulate internal and external stimuli, signal transduction pathways, and stress responses. Plant hormones are essential for growth and development during water deficit stress. Water stress activates plant growth regulators ABA, JA, SA, and ET [169].

### 6.1. Exogenous ABA and JA in Vegetable Drought Tolerance

The hormone ABA regulates various physiological processes. Osmotic stress, caused by low water availability, triggers ABA production and plant adaptation mechanisms [170]. The synthesis of ABA begins in the plastids upon receiving stress signals at the plasma membrane, and it takes place in the cytoplasm, excluding xanthorin transition. A significant portion of ABA is produced in the roots and then transported to the upper parts of the plant through vascular tissues [171,172]. ABA is crucial in activating stress-responsive genes under various conditions, including osmotic stress [173,174]. Several receptors for ABA have been identified in the cytosol, plasma membrane, chloroplast envelope, and nucleus. In plants with low ABA levels, protein phosphatase 2C (PP2C) inhibits the activity of non-fermenting sucrose 1-linked protein kinase 2 (SnRK2) proteins through dephosphorylation [175]. A plethora of studies noted increased drought tolerance regulating stress-related genes through ABA [176]. The overexpression of ABA-induced gene GhCBF3 leads to higher drought tolerance in transgenic lines than in wild-type plants by maintaining chlorophyll, root weight, and proline [177,178]. ABA is involved in leaf abscission and drought stress in plants [179]. It plays a primary and critical role in developmental and physiological activities, including seed dormancy, maintenance of tumor cells, stomatal opening, embryo morphogenesis, and the production of fats and stored proteins. ABA influences the expression of protein-coding genes [180]. It is also required for root development and structural changes in nitrogen-deficient plants. Enzymes such as dehydrins, osmoprotectants, and protective proteins are produced under the influence of ABA. ABA serves two functions in drought stress: maintaining water balance in cells by regulating guard cells and expressing genes that produce proteins for dehydration tolerance.

A study reported that lower levels of ABA in leaves resulted in greater drought tolerance than leaves with higher proline levels [181]. During the drying process, soil moisture levels play a more critical role than leaf water levels, primarily influenced by the production of ABA in the roots [182,183]. In drought stress, the phytohormone ABA regulates morpho-physiology and biochemistry via stomatal closure. Stomatal closure is the most crucial and effective response to ABA during drought [184]. However, ABA functions as a signaling molecule in olive plants to aid their adaptation to drought [185]. Previous reports involving the application of exogenous ABA on leaves have shown that this application induces several adaptive changes in response to water scarcity. This includes the enhanced activity of antioxidant enzymes such as GR, SOD, APX, and CAT in tomato plants (*Solanum lycopersicum* L.) [186]. In addition, the exogenous application of ABA has been found to reduce reactive oxygen species (ROS) levels and enhance cell membrane stability (CMS), aiding in the recovery of plants after experiencing stress [187]. ABA is linked to the up-regulation of cuticle-related genes, leading to increased cuticular wax production observed in tomatoes [188] and cucumbers [189]. This elevation aids in reducing non-stomatal transpiration and enhances tolerance to water deficit conditions. Several studies have demonstrated that spraying plants with exogenous ABA can improve stress tolerance in various crop species. However, there is a significant lack of research investigating the responses of different soybean (*Glycine max* L.) varieties to drought stress using exogenous ABA and fluoridone, an ABA synthesis inhibitor [190,191]. During drought stress, ABA significantly increases the activities of SOD and POD, but these activities decline significantly after re-watering. Under drought stress conditions, ABA priming notably enhances the relative water content in wheat cultivars [192]. ABA acts as a primary stress sensor in plant drought response pathways, enabling plants to improve their response to desiccation. The increase in ABA concentration corresponds to the accumulation of lycopene and carotene in fruits [193,194].

JA is a phytohormone that exists in plants, and its active derivatives are referred to as jasmonates. It is a defense mechanism against biotic and abiotic stressors [195]. Additionally, JA has been associated with enhancements in root structure, pollen production, tendril coiling, and fruit ripening in numerous plant species [196]. Exogenous JA has been shown to enhance plant performance and regulate stomatal dynamics under dry conditions. Extensive research has been conducted on the signaling pathway and production of JA [196,197]. In the absence of water, JAZ proteins undergo degradation, activating transcription factors such as MYC2. These transcription factors then stimulate the up-regulation of stress tolerance genes [198]. Plant hormones typically do not function through single pathways but rather interact with each other at different stages to regulate both environmental and developmental processes. Signal transduction mechanisms in plants are intricate and coordinate a complex array of events to enable adaptation to challenging environments. Jasmonates (JAs) are complex phytohormones generated through the breakdown of cell membrane lipids in various plant species [199]. These JAs, considered plant growth regulators, are present in almost all plant tissues. Jasmonates also interact with other phytohormones to regulate plant growth, development, and response to biotic and abiotic stimuli. JAs have diverse effects on seed dormancy and germination. Under water-stressed conditions, treatment with JAs has negatively affected seed germination in several vegetable species, including *Solanum Lycopersicon* [200,201]. However, our understanding of the precise impact of JAs on seed germination under water deficit conditions remains limited.

### 6.2. Exogenous SA and ET in Vegetable Drought Stress Tolerance

SA is a phenolic molecule produced through secondary metabolism and involved in various biological processes, including CO_2_ assimilation, antioxidation, stomatal regulation, and photosynthesis [202,203]. A series of studies have been conducted to identify the role of SA in abiotic and biotic stress; limited evidence exists regarding its impact on drought stress. Nevertheless, several studies propose that SA may contribute to drought stress response by modulating the expression of drought-related genes and influencing stomatal aperture. The effects of SA on drought tolerance or sensitivity depend on the quantity of SA applied [204,205]. In the case of cpr5 and acd6 mutants, an increase in SA accumulation and reduced stomatal conductance were observed, indicating enhanced tolerance to drought stress.

Additionally, priming seedlings with SA revealed numerous vital proteins involved in drought stress physiology and metabolism [206]. These proteins encompass carbohydrate metabolism, photosynthesis, antistress proteins, and the signaling cascade. As a result, the primed seedlings exhibited improved growth and increased tolerance to drought [207,208]. Furthermore, applying SA from external sources has been found to augment plants’ resilience to drought. Overexpression of *CBP60g*, a transcriptional regulator of SA biosynthesis, led to heightened sensitivity to ABA, elevated SA accumulation, and robust resistance to drought in plants [209]. Research by [210] suggests that SA regulates proline production and strengthens the cellular redox state in *Brassica rapa* L. plants.

Additionally, high levels of SA and the presence of the siz1 mutant, which affects the function of SUMO E3 ligase SIZ1, were found to reduce light-induced stomatal opening in plants, thereby minimizing water loss and conferring drought resistance [211]. However, despite the extensive exploration of ethylene’s diverse functions, its involvement in drought stress response has received relatively less investigation. Recent research on wheat genotypes exposed to mild drought stress revealed that the tolerant group exhibited higher dry shoot weight than the sensitive group, which correlated with increased ethylene levels [212,213]. Interestingly, studies examining the effect of ethylene on stomatal closure have yielded conflicting results. Mutant eto1 that exhibits elevated ethylene accumulation displayed slower stomatal closure under drought stress than in control plants, despite ethylene being generally associated with improved stomatal closure in guard cells [214]. Conversely, the rice etol1 mutant, characterized by higher ethylene accumulation, exhibited enhanced drought tolerance compared to OsETOL1 plants susceptible to drought stress treatment. Additionally, modifying genes within the ethylene signaling pathway has led to generating drought-tolerant transgenic plants [215,216]. These findings emphasize the importance of comprehending and leveraging ethylene signaling under abiotic stressors to elucidate and harness drought stress tolerance-related traits in crops.

## 7. Conclusions

In summary, the study of drought stress tolerance in vegetables has yielded crucial insights into the intricate mechanisms governing a plant’s ability to endure water scarcity. Analyzing structural features, gene pathways, and exogenous hormone impacts has deepened our comprehension of plant responses to drought stress. Structural attributes like cuticle thickness, stomatal density, and root architecture play pivotal roles in managing water regulation, directly influencing a plant’s resilience to drought. Key gene pathways controlling stress perception, signal transduction, and activation of stress-responsive genes have been unveiled, with transcription factors like DREB and bZIP emerging as pivotal drivers of adaptive plant reactions. The interplay between exogenous hormones and plants under drought stress reveals a complex dynamic that can either enhance or hinder a plant’s drought-coping mechanisms. Notably, the significance of ABA is evident as it aids stomatal closure, reduces transpiration, and initiates molecular cascades that enhance stress tolerance.

This comprehensive knowledge can be harnessed by researchers and agricultural experts to devise innovative strategies for bolstering drought stress tolerance in vegetable crops. Approaches may span from traditional breeding to biotechnological interventions, including genetic manipulation and targeted hormone treatments. By optimizing structural traits, activating stress-responsive genes, and modulating hormone interactions, agricultural productivity and sustainability can be advanced in water-scarce regions. Given the escalating impact of climate change and erratic precipitation, the insights derived from studying vegetable drought tolerance mechanisms offer broad implications. Beyond enhancing our fundamental grasp of plant biology, these insights furnish practical tools to ensure food security and counteract water-related challenges in global agriculture. The continual pursuit of research in this field promises further breakthroughs, propelling the development of robust crop varieties and cultivating a more sustainable agricultural future.

## Data Availability

Not applicable.

## Abbreviations

ROS	Reactive oxygen species
ABA	Abscisic acid
JA	Jasmonic acid
RWC	Relative water content
OA	Osmotic adjustment
WUE	Water-use efficiency
SOD	Superoxide dismutase
POD	Peroxidase
GR	Glutathione reductase
APX	Ascorbate peroxidase
CAT	Catalase
IP3	Inositol trisphosphate
MDA	Malondialdehyde
Ca^2+^	Calcium ion
cADPR	Cyclic adenosine diphosphate ribose
CaM	Calmodulin
CBLs	Calcineurin B-like proteins
MAPK	Mitogen-activated protein kinase
P5CS	△-pyrroline-5-carboxylate synthetase
P5CR	Pyrroline-5-carboxylate reductase
ProDH	Proline dehydrogenase
Glu	Glutamic acid
Orn	Ornithine
P-MME	Phosphate-monomethyl-ethanolamine
CMO	Choline monoocygenase
